# A Fulminant Case of Refractory Sarcoidosis Triggered by Infliximab-Neutralizing Antibodies

**DOI:** 10.7759/cureus.91629

**Published:** 2025-09-04

**Authors:** Mohamed Ibraheem, Fahed Owda, Shatha Mallah, Hamza Saleem, Ahmed Nabhan, Jehad Azar

**Affiliations:** 1 Internal Medicine, Transplant Center, Mayo Clinic, Phoenix, USA; 2 Internal Medicine, An-Najah National University, Nablus, PSE; 3 Internal Medicine, Mayo Clinic, Phoenix, USA; 4 Pulmonary Medicine, Mayo Clinic, Phoenix, USA

**Keywords:** cardiac sarcoidosis, infliximab, low-dose methotrexate, neutralizing antibodies, pulmonary sarcoidosis

## Abstract

Sarcoidosis is a multisystem granulomatous disorder, commonly involving the lungs, heart, and skin. Despite recent dramatic advancements in the field, the exact cause remains an enigma; yet, we believe the pathogenesis represents an interplay between environmental, infectious, and genetic factors. An abnormal T regulatory response, in parallel with an enhanced Th1 response, results in the release of cytokines, including IL-2, IL-12, IL-18, tumor necrosis factor-alpha (TNF-α), and interferon-gamma (IFN-γ), which initiates granuloma formation. Meanwhile, enhanced Th17 production stimulates the Janus kinase-signal transducer and activator of transcription (JAK-STAT) signaling pathway, ultimately leading to granuloma expansion. The recent evolution in understanding the immunologic pathogenesis of sarcoidosis has led to the introduction of advanced treatment modalities for refractory cases resistant to steroids, methotrexate, and leflunomide, which are often used as first-line treatments. TNF-α inhibitors are considered a pioneering treatment with promising effects in advanced sarcoidosis, especially for neurosarcoidosis, cardiac, cutaneous, and refractory pulmonary cases. We present a very rare case of refractory pulmonary sarcoidosis that progressed to fulminant cardiac sarcoidosis, manifesting as heart failure and malignant arrhythmia despite high-dose infliximab treatment. This refractory course was attributed to the emergence of infliximab-neutralizing antibodies, which rendered infliximab ineffective. This case raises awareness about a preventable progression of disease that may otherwise have a fatal outcome, mandating strict follow-up for the emergence of neutralizing antibodies and the need for concomitant low-dose methotrexate, prednisone, and/or azathioprine to prevent such life-threatening sequelae.

## Introduction

Sarcoidosis is a chronic inflammatory disease characterized by non-caseating granulomas affecting multiple organs, most commonly the lungs and lymphatic system [1,2]. While many patients respond to corticosteroids and immunosuppressive agents, a subset remains refractory and may require biologic therapies such as tumor necrosis factor-alpha (TNF-α) inhibitors. However, the development of neutralizing antibodies can reduce the efficacy of these agents, complicating management. Recognizing and addressing immunogenicity is essential for optimizing outcomes in patients with complex or treatment-resistant sarcoidosis [3].

This case underscores the importance of monitoring of infliximab-neutralizing antibodies development in patients with refractory sarcoidosis treated with TNF-α inhibitors. Early recognition of immunogenicity and timely adjustment of therapy, including switching to a different biologic agent, can restore clinical response and improve patient outcomes. Multidisciplinary management and individualized treatment strategies remain essential for addressing the complexities of refractory sarcoidosis [4,5].

## Case presentation

A male patient in his 70s, with a past medical history of hypertension, dyslipidemia, and biopsy-proven sarcoidosis with multisystem involvement, was admitted to the medical intensive care unit (MICU) with acute hypoxic respiratory failure. He has carried the diagnosis of sarcoidosis for the last five years, which was initially suspected based on a computed tomography (CT) chest that was done for chronic cough and showed bilateral symmetrical hilar and mediastinal lymph nodes enlargement. Bronchoscopy with endobronchial ultrasound-guided transbronchial needle aspiration (EBUS-TBNA) showed a non-caseating granuloma, with negative broad infectious evaluation, including fungal and mycobacterial cultures. Flow cytometry for lymphoma and lymphoproliferative disease was also negative, and so was the cytology. He was also found to have an elevated angiotensin-converting enzyme (ACE) level at 78 U/L, elevated 1,25-dihydroxy vitamin D at 71 ng/mL, high calcium at 11.9 mg/dL, and low 25-hydroxy vitamin D at 12 ng/mL, making the diagnosis of sarcoidosis (Table 1).

**Table 1:** Laboratory and cardiopulmonary parameters supporting the diagnosis of overlap sarcoidosis and IPF with cardiac involvement. This table summarizes relevant laboratory, pulmonary function, and hemodynamic findings that contributed to the diagnosis of overlapping sarcoidosis and IPF, complicated by cardiac involvement and pulmonary hypertension. PH: pulmonary hypertension; IPF: idiopathic pulmonary fibrosis

Parameters	Obtained value	Reference range	Clinical interpretation
Angiotensin-converting enzyme (ACE)	78 U/L	8-52 U/L	Elevated - supports diagnosis of sarcoidosis
1,25-Dihydroxy vitamin D	71 ng/mL	15-60 ng/mL	Elevated - consistent with sarcoidosis activity
Calcium	11.9 mg/dL	8.5-10.5 mg/dL	Elevated - hypercalcemia, common in sarcoidosis
25-Hydroxy vitamin D	12 ng/mL	20-50 ng/mL	Low - vitamin D deficiency
Forced vital capacity (FVC)	58% predicted	>80% predicted	Elevated - restrictive lung pattern
Diffusion capacity for carbon monoxide (DLCO)	51% predicted	>80% predicted	Moderately reduced - impaired gas exchange
Ejection fraction (EF)	45%	50-70%	Reduced - new-onset systolic heart failure
Right atrial pressure (RAP)	12 mmHg	2-8 mmHg	Elevated - poor prognosis for PH
Mean pulmonary artery pressure (mPAP)	35 mmHg	<20 mmHg	Elevated - pulmonary hypertension
Pulmonary artery wedge pressure (PAWP)	11 mmHg	6-12 mmHg	Normal
Cardiac output (Fick method)	4.5 L/min	4-8 L/min	Normal
Cardiac index	2.3 L/min/m²	2.5-4.0 L/min/m²	Low to normal - borderline perfusion
Pulmonary vascular resistance (PVR)	5.3 Wood units	<2 Wood units	Elevated - precapillary pulmonary hypertension

**Figure 1:**
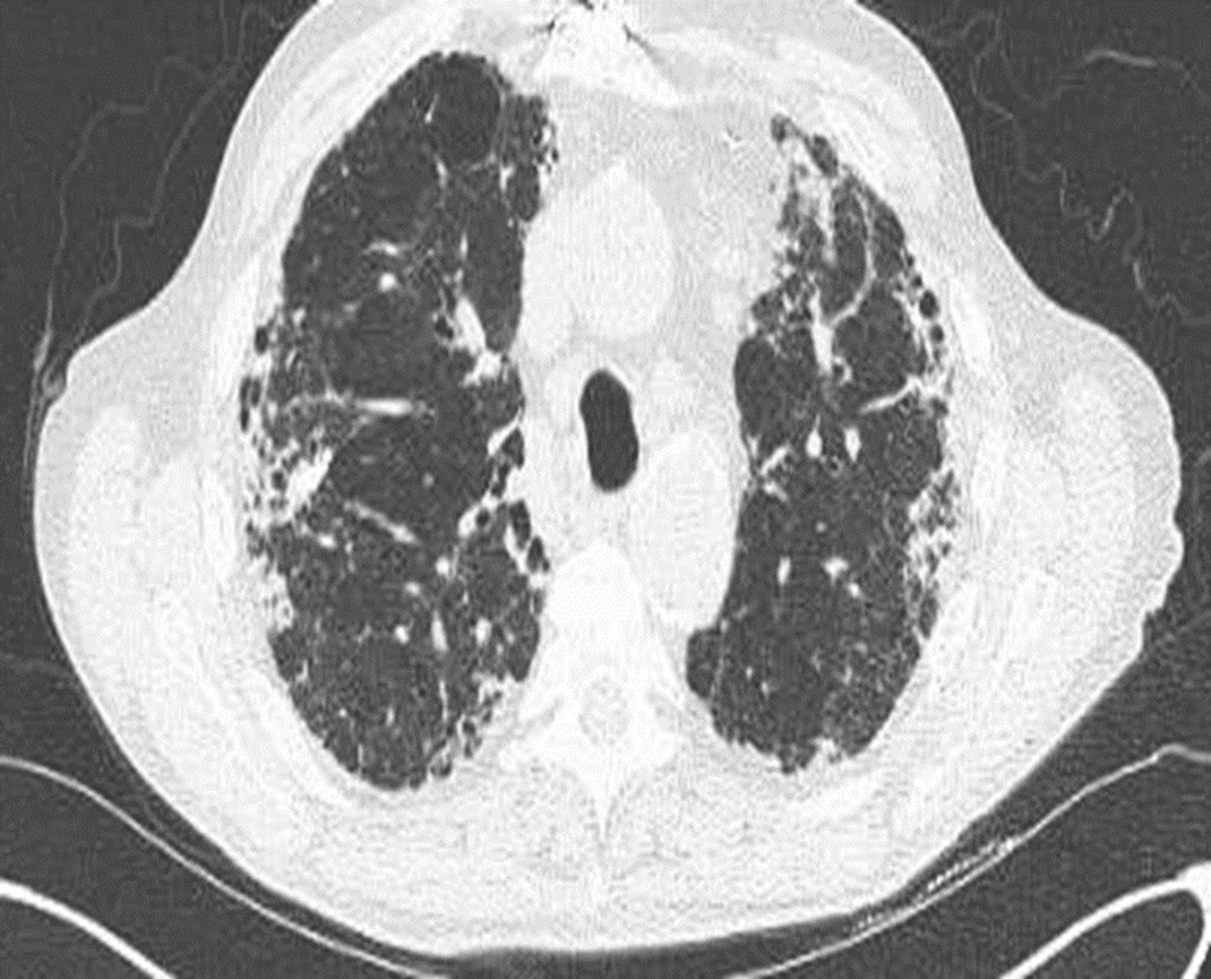
Axial CT chest showing lower lobe predominant fibrotic changes. Axial high-resolution CT image of the chest with no contrast shows reticulation with peripheral distribution involving the subpleural space more predominant in the lower lung field, with traction bronchiectasis, traction bronchioloectasis, honeycombing, architectural distortion, and volume loss diagnostic of usual interstitial pneumonia pattern (sarcoidosis idiopathic pulmonary fibrosis overlap syndrome).

**Figure 2:**
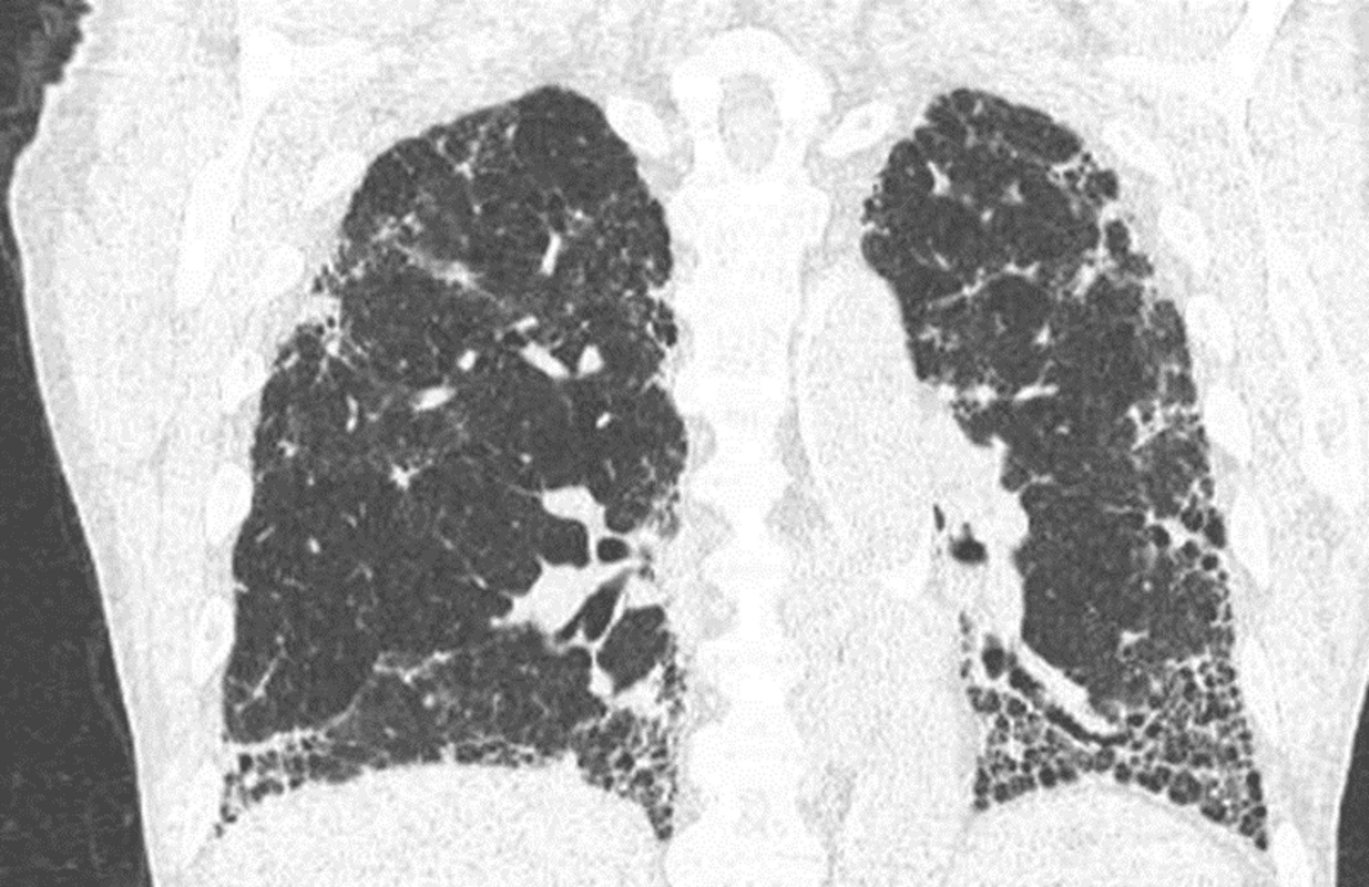
Coronal CT chest with no contrast highlighting lower lobe-predominant UIP features. Coronal high-resolution CT image of the chest showing a fibrotic change of usual interstitial pneumonitis pattern; peripheral reticulation, interseptal thickening, traction bronchiectasis, traction bronchiolectasis, architectural distortion, honeycombing, and volume loss. UIP: usual interstitial pneumonia

**Figure 3:**
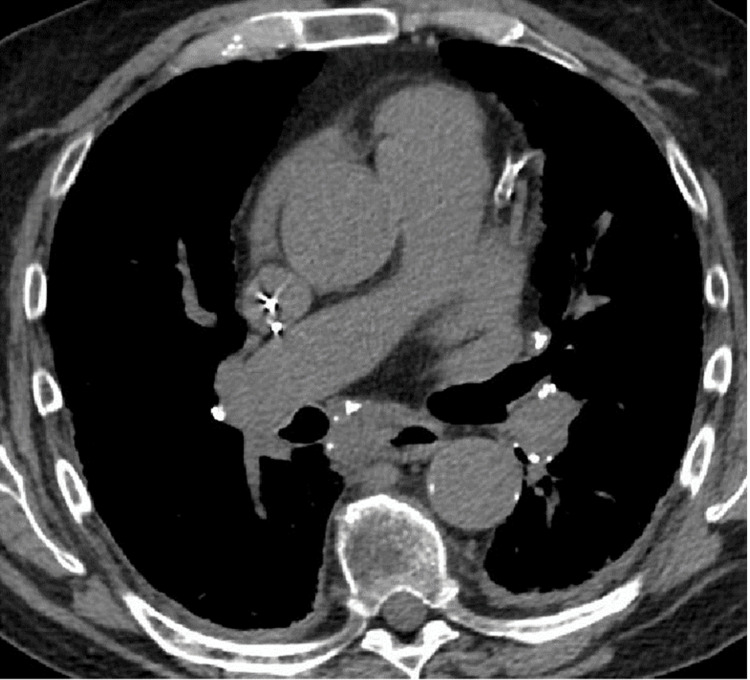
CT chest demonstrating calcified hilar lymphadenopathy. CT chest without contrast (soft tissue window) reveals bulky, symmetrical calcified hilar lymphadenopathy, consistent with sarcoidosis radiological stage I.

**Figure 4:**
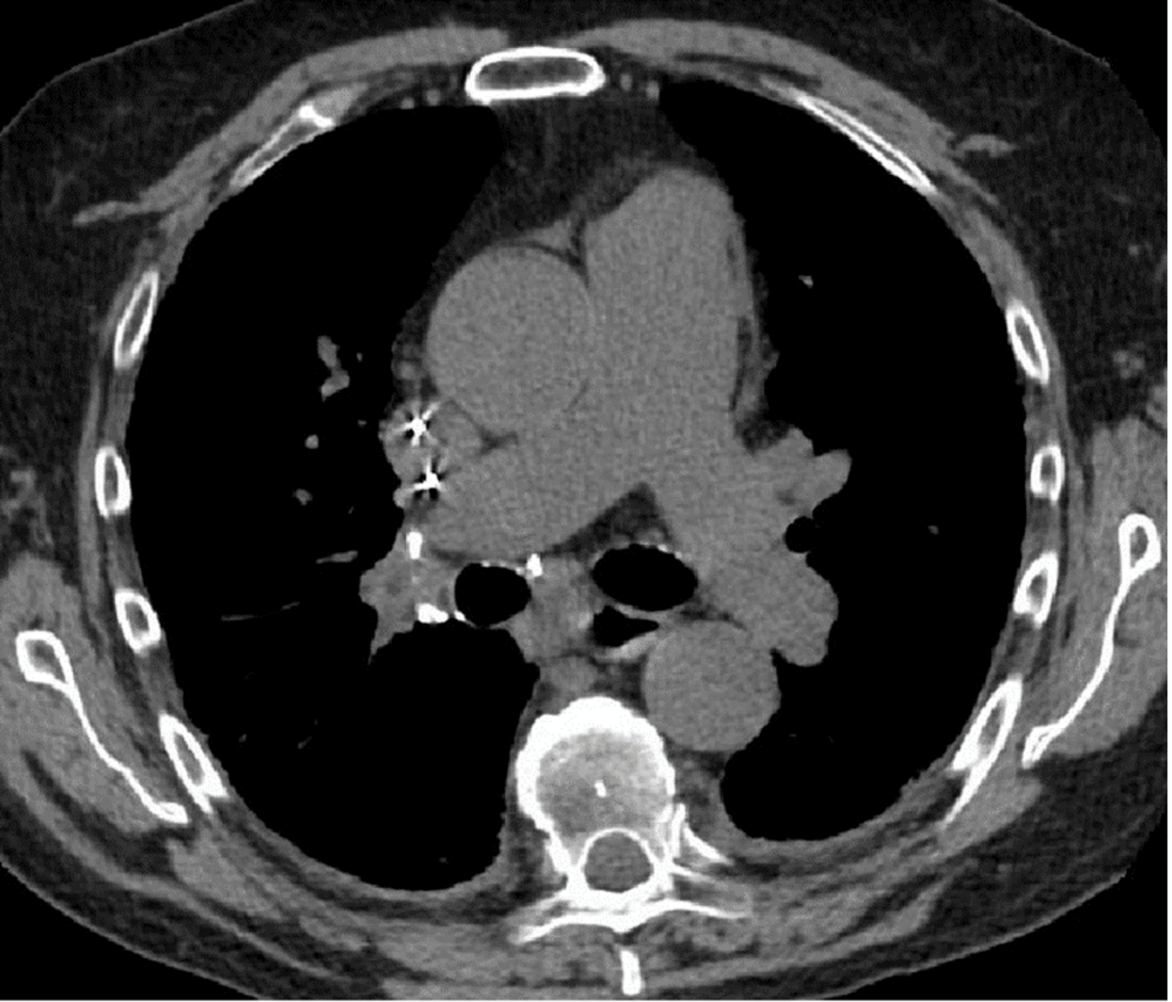
CT chest demonstrating calcified mediastinal lymphadenopathy. CT chest without contrast (soft tissue window) shows bulky, symmetrical calcified mediastinal lymphadenopathy, consistent with long-standing sarcoidosis radiological stage I.

A few months later, his course became refractory to the typical first- and second-line treatments and was ultimately diagnosed with neurosarcoidosis with thoracic and cervical spine involvement, for which he was started on infliximab 5 mg/kg every four weeks (Q4W) and low-dose methotrexate at 10 mg, after a short course of systemic steroids. Over the years, his disease had been controlled with no respiratory or cardiovascular complaints, nor did he have any other symptoms suggesting other organs. A year before his current presentation, he developed a new-onset progressive exertional dyspnea, dry cough, and fatigue. CT chest showed diffuse ground-glass opacities (GGO), inter-septal thickening, patchy consolidation, and stable symmetrical hilar and mediastinal lymphadenopathy. Methotrexate was stopped for suspected methotrexate-induced lung injury, and he was maintained on monotherapy with infliximab. Despite holding methotrexate, there was no improvement in his exertional dyspnea or cough. A repeated chest CT showed resolution of previously noted ground-glass opacities (GGO) and consolidation. However, new-onset fibrotic changes - predominantly in the lower lobes - were observed, consistent with a probable usual interstitial pneumonia (UIP) pattern. These included subpleural reticulation, traction bronchiectasis, traction bronchiolectasis, honeycombing, volume loss, and architectural distortion (Figures 1, 2). Additionally, stable, symmetrical calcified hilar and mediastinal lymphadenopathy were noted (Figures 3, 4). His CT chest shows bulky symmetrical calcified hilar and mediastinal lymphadenopathy consistent with sarcoidosis radiological stage I (Figures 3, 4).

Pulmonary function tests revealed restrictive lung disease with forced vital capacity (FVC) reduced to 58% predicted, and moderately reduced diffusion capacity for carbon monoxide (DLCO) at 51% predicted. He underwent bronchoscopy with transbronchial biopsy and genomic classifier, Envisia study, which returned positive. The biopsy results were consistent with UIP with heterogeneous patchy areas of subpleural fibrotic foci, traction bronchiectasis, bronchioloectasis, and honeycombing. Subsequently, he was diagnosed with overlap syndrome of pulmonary sarcoidosis and idiopathic pulmonary fibrosis (IPF), given a negative evaluation for his UIP pattern evaluation; ultimately, he was started on nintedanib 150 mg BID.

A year later, he was admitted with hypoxic respiratory failure and generalized anasarca. His echocardiography showed new-onset heart failure with reduced ejection fraction (EF) from 63% to 45%, new-onset right ventricular (RV) failure, severe tricuspid regurgitation (TR), RV systolic pressure of 75 mmHg, and new-onset pericardial effusion. Cardiac MRI revealed late gadolinium enhancement in a distribution that fits the diagnosis of cardiac sarcoidosis; moreover, cardiac PET scan showed matched perfusion/fluorodeoxyglucose (FDG) avid defects correlating with areas of FDG uptake consistent with active cardiac sarcoidosis. Right heart catheterization (RHC) revealed right atrial pressure of 12 mmHg, mean pulmonary artery pressure (MPAP) of 35 mmHg, pulmonary artery wedge pressure (PAWP) of 11 mmHg, cardiac output (CO) of 4.5 L/min by the Fick method, cardiac index of 2.3 L/min/m², and pulmonary vascular resistance (PVR) of 5.3 Wood units. Evaluation for group 4 pulmonary hypertension was negative, confirming the diagnosis of group 3 pulmonary hypertension in the setting of interstitial lung disease. Infliximab activity was evaluated and found to be positive for neutralizing antibodies and undetectable infliximab levels, confirming infliximab immunogenicity and explaining his presentation with cardiac sarcoidosis despite being on infliximab. Unfortunately, his admission was eventful for a life-threatening arrhythmia sustained with ventricular tachycardia, for which an implantable cardioverter-defibrillator (ICD) was placed. He was started on prednisone 60 mg with slow taper, leflunomide 20 mg, and infliximab was switched to adalimumab. He was also started on guideline-directed medical therapy (GDMT) for his heart failure with normalization of his EF. Ultimately, he was started on inhaled treprostinil for group 3 pulmonary hypertension. His clinical pictures improved back to his baseline, and he was discharged on home oxygen at 4 liters per minute (LPM).

Outcome and follow-up

Upon six-week post-discharge outpatient clinic follow-up, he showed signs of recovery. Clinically, he was euvolemic with no signs of heart failure. Prednisone was tapered off, and he was maintained on leflunomide and adalimumab 40 mg weekly.

## Discussion

Sarcoidosis, a systemic granulomatous disease with an unknown etiology, primarily targets the lungs and lymphatics but can affect any organ or system. While some cases may enter remission spontaneously with or without treatment, approximately a quarter of patients experience a chronic course [1]. Sarcoidosis manifests as non-caseating granulomas, hilar lymphadenopathy, and lung reticulonodular opacities with an unpredictable clinical course [2]. From asymptomatic cases with incidental radiographic findings to refractory chronic respiratory disease, its pulmonary presentation spans a spectrum, with respiratory failure being the leading cause of mortality [3]. Our patient presented with acute hypoxemic respiratory failure, pulmonary hypertension, lung fibrosis, cardiac sarcoidosis, and neurosarcoidosis, necessitating an intensive evaluation and management due to his poor prognosis [3].

Corticosteroids are the first-line treatment for sarcoidosis, but the potential for cumulative toxicity limits their extended use [1,4]. While the vast majority of cases respond well to glucocorticoid therapy, some patients require second-line steroid-sparing immunosuppressive agents, such as methotrexate and azathioprine. Refractory sarcoidosis, comprising up to 10%, is linked to heightened morbidity and mortality and is characterized by failure to achieve clinical remission despite corticosteroids and immunosuppressive therapy [4,5]. Infliximab and adalimumab, monoclonal antibodies tailored to target tumor necrosis factor-alpha (TNF-α), a pivotal cytokine in sarcoidosis, constitute third-line therapy and have demonstrated effectiveness in treating refractory sarcoidosis [5,6]. Among these anti-TNF-α agents, infliximab has garnered the most extensive research and is commonly used [7]. For patients with sarcoidosis who are resistant to TNF inhibitors, recent proposals have introduced fourth-line therapies, including JAK inhibitors and other biologics, such as rituximab and tocilizumab [8,9].

While all biological drugs trigger immunogenicity to some extent, the degree varies. This discrepancy is partly attributed to structural variations among anti-TNF agents, with lower immunogenicity rates associated with greater humanization of the molecules. Among biologics, infliximab exhibits the highest immunogenicity rate, particularly due to its chimeric nature, with anti-drug antibodies forming against the F(ab)_2_ fragment of the anti-TNF IgG molecule [10-12]. The formation of anti-infliximab antibodies could impact its pharmacokinetics, leading to increased clearance and reduced serum concentrations. Studies in conditions other than sarcoidosis have indicated that patients with positive antibody tests against infliximab face an elevated risk of allergic reactions and a threefold higher likelihood of losing clinical response [10,11]. In our case, infliximab-neutralizing antibody formation led to a fulminant course with respiratory failure, new-onset cardiac sarcoidosis presenting with biventricular failure, and pulmonary hypertension.

In the management of neurosarcoidosis, corticosteroids also serve as the initial treatment; however, about 25% of patients require escalation to second- or third-line therapies. In cases of inadequate response to glucocorticoids or significant concern regarding their toxicity, early intervention with TNF inhibitors is increasingly considered [13]. A multinational study supports the efficacy of infliximab in addressing neurosarcoidosis. Additionally, promising evidence indicates that alternative anti-TNF agents, such as adalimumab, may also offer effectiveness in managing neurosarcoidosis, which affects roughly 5-10% of sarcoidosis patients [14,15].

The role of concurrent immunosuppression, such as methotrexate (MTX), in reducing the formation of anti-drug antibodies (ADAs) for TNF inhibitors remains unclear in sarcoidosis, with its application often based on studies conducted in rheumatoid arthritis and Crohn’s disease [10,16]. The immunosuppressive effects of MTX on T and B cells may provide insight into its pharmacological role in reducing ADAs targeting anti-TNF agents [17]. Determining the optimal dosage and administration route of MTX to enhance anti-TNF levels and prevent ADA formation remains a subject of investigation. Studies have shown that oral MTX is as effective as parenteral administration in enhancing clinical outcomes when combined with anti-TNFs [18,19]. The lower dosage, typically up to 10 mg, of oral MTX has the best bioavailability when compared to high doses. This underscores the reason for the efficacy of oral MTX 10-12.5 mg/week in reducing ADA formation and boosting anti-TNF levels in rheumatologic diseases and inflammatory bowel disease (IBD) [19]. Our patient was initially maintained on low-dose oral MTX and infliximab (IFX); however, stopping methotrexate due to concerns for pulmonary toxicity ultimately led to ADA formation and treatment failure, resulting in a devastating outcome. Our case sheds light on such rare and preventable phenomena; concomitant treatment with low-dose methotrexate, azathioprine, or prednisone can prevent an otherwise fatal outcome. Further research to better understand the pathogenesis and proper management guidelines is needed.

In rheumatoid arthritis, concurrent use of MTX has been associated with reduced immunogenicity in adalimumab treatment. Additionally, one study revealed the impact of MTX on adalimumab's pharmacokinetics in patients with rheumatoid arthritis, with those receiving concomitant MTX showing higher median adalimumab concentrations (7.4 μg/mL) compared to those without (4.1 μg/mL). A meta-analysis conducted by Thomas et al. revealed a statistically significant contrast in the incidence of ADAs between IFX and ADL. IFX displayed a higher frequency of ADA occurrence (25.3%) compared to ADL, a fully humanized monoclonal antibody (14.1%). This variation accounts for the broader usage of immunomodulators alongside IFX compared to other anti-TNF agents [20].

Transitioning to ADL in patients experiencing intolerance to IFX due to allergic reactions, ADA formation, or severe adverse events was supported by a randomized controlled trial [21]. Additionally, a study published by Crommelin et al. showed that among 142 patients initially treated with infliximab, 18 discontinued treatment due to ADA formation or severe adverse events, subsequently transitioning to adalimumab therapy. Following 12 months of treatment, organ function improved in 39% of patients and remained stable in 33%. Our patient maintained clinical recovery after transitioning to ADL, proving its effectiveness in patients resistant to IFX [20,21].

In a case report by Vincent et al., a complete reversal of anti-IFX antibodies was observed upon the introduction of MTX in a 50-year-old woman with severe plaque psoriasis. This observation suggests that switching to another TNF inhibitor may be unwarranted, as the addition of MTX alone achieved persistent remission. However, further studies are necessary to validate these findings. Compared to other autoimmune diseases, immunogenicity to biologics in refractory sarcoidosis remains a relatively underexplored area of research, lacking clear guidelines on management. Despite affecting a smaller number of patients, addressing this issue should be prioritized in future trials. Finding effective immunosuppressant options to prevent ADA formation is crucial in preventing sarcoidosis flares and averting fatal complications [22].

## Conclusions

Infliximab is a chimeric monoclonal antibody directed against tumor necrosis factor-alpha (TNF-α) and is widely employed in the management of conditions, such as sarcoidosis. Although it demonstrates considerable efficacy, its chimeric design predisposes patients to the development of neutralizing antibodies, which may attenuate therapeutic benefit over time. By contrast, adalimumab, a fully human monoclonal antibody, is associated with a comparatively lower risk of immunogenicity. In this case, the patient experienced a loss of clinical response to infliximab, underscoring the need for vigilant monitoring, including assessment of trough drug levels and detection of anti-drug antibodies. This clinical course further highlights the importance of concomitant use of immunosuppressive agents to mitigate antibody formation. The selection of an immunosuppressive agent should be individualized; however, methotrexate at a dose of 10-15 mg weekly is frequently employed, supported by evidence largely drawn from studies in inflammatory bowel disease. Early recognition of diminished drug activity and timely adjustment of therapy are critical in the management of patients with severe or multisystem disease. Moreover, further investigation is warranted to better elucidate the potential role of immunosuppression, particularly with prednisone and methotrexate, in facilitating the clearance of TNF-α inhibitor-neutral antibodies once they have developed.
